# An integrated surveillance network for antimicrobial resistance, India

**DOI:** 10.2471/BLT.20.284406

**Published:** 2021-06-01

**Authors:** Sonam Vijay, Monica Sharma, Jyoti Misri, BR Shome, Balaji Veeraraghavan, Pallab Ray, VC Ohri, Kamini Walia

**Affiliations:** a Division of Epidemiology and Communicable Diseases, Indian Council of Medical Research, Ansari Nagar, New Delhi, 110029, India.; b Division of Animal Science, Indian Council of Agricultural Research, New Delhi, India.; c Microbial Pathogenesis and Pathogen Diversity Laboratory, Indian Council of Agricultural Research–National Institute of Veterinary Epidemiology and Disease Informatics, Bengaluru, India.; d Department of Clinical Microbiology, Christian Medical College, Vellore, India.; e Department of Medical Microbiology, Postgraduate Institute of Medical Education and Research, Chandigarh, India.

## Abstract

**Objective:**

To assess the preparedness of veterinary laboratories in India to participate in an integrated antimicrobial resistance surveillance network and to address gaps in provision identified.

**Methods:**

The Indian Council of Medical Research and the Indian Council of Agricultural Research collaborated: (i) to select eight nationally representative veterinary microbiology laboratories whose capacity for participating in an integrated antimicrobial resistance surveillance network would be assessed using a standardized tool; (ii) to identify gaps in provision from the assessment findings; and (iii) to develop a plan, and take the necessary steps to address these gaps in consultation with participating organizations.

**Findings:**

The main gaps in provision identified were: (i) a lack of dedicated funding for antimicrobial resistance surveillance; (ii) the absence of standard guidelines for antimicrobial susceptibility testing; (iii) a shortage of reference strains for testing and quality assurance; and (iv) the absence of mechanisms for sharing data. We addressed these gaps by creating a veterinary standard operating procedure for antimicrobial susceptibility testing, by carrying out a validation exercise to identify problems with implementing the procedure and by conducting capacity-building workshops for veterinary laboratories.

**Conclusion:**

Antimicrobial resistance surveillance networks depend on the availability of accurate, quality-controlled testing. The challenges identified in creating an integrated surveillance network for India can be overcome by developing a comprehensive plan for improving laboratory capacity in human, veterinary and environmental sectors that is supported by the necessary funds. The study’s findings may provide guidance for other low- and middle-income countries planning to develop a similar network.

## Résumé

**Objectif:**

Évaluer le degré de préparation des laboratoires vétérinaires en Inde dans l'optique d'une participation à un réseau de surveillance intégré pour la résistance aux antimicrobiens, et combler les lacunes identifiées dans le système.

**Méthodes:**

Le Conseil indien de la recherche médicale et le Conseil indien de la recherche agronomique ont travaillé ensemble afin de: (i) sélectionner huit laboratoires de microbiologie vétérinaire représentatifs à l'échelle nationale, dont la capacité de participation à un tel réseau serait évaluée grâce à un outil standardisé; (ii) déterminer quelles sont les faiblesses du système à partir des résultats de l'évaluation; et, enfin, (iii) développer un plan et prendre les mesures nécessaires pour remédier à ces faiblesses, en concertation avec les organismes participants.

**Résultats:**

Les principales lacunes observées dans le système étaient: (i) l'absence de fonds dédiés à la surveillance de la résistance aux antimicrobiens; (ii) l'absence de directives types pour le test de sensibilité aux antimicrobiens; (iii) le manque de souches de référence pour les tests et l'assurance qualité; et, enfin, (iv) l'absence de mécanismes de partage de données. Nous avons comblé ces lacunes en créant une procédure opérationnelle standardisée à usage vétérinaire pour le test de sensibilité aux antimicrobiens, en menant un exercice de validation afin d'identifier les problèmes rencontrés lors de la mise en œuvre de la procédure, et en organisant des ateliers de renforcement des capacités pour les laboratoires vétérinaires.

**Conclusion:**

Les réseaux de surveillance pour la résistance aux antimicrobiens dépendent de l'existence ou non de tests précis faisant l'objet de contrôles de qualité. Les défis que pose la création d'un réseau de surveillance intégré en Inde peuvent être relevés grâce à l'élaboration d'un plan global d'amélioration des capacités humaines, vétérinaires et environnementales des laboratoires en y injectant les fonds requis. Les résultats de cette étude peuvent servir d'orientation pour d'autres pays à faibles et moyens revenus qui prévoient d'instaurer un réseau similaire.

## Resumen

**Objetivo:**

Evaluar la preparación de los laboratorios veterinarios de la India para participar en una red integrada de vigilancia de la resistencia a los antimicrobianos y subsanar las deficiencias detectadas en la prestación.

**Métodos:**

El Consejo Indio de Investigación Médica y el Consejo Indio de Investigación Agrícola colaboraron: (i) para seleccionar ocho laboratorios de microbiología veterinaria representativos a nivel nacional cuya capacidad para participar en una red integrada de vigilancia de la resistencia a los antimicrobianos sería evaluada mediante una herramienta estandarizada; (ii) para identificar las lagunas en la prestación a partir de los resultados de la evaluación; y (iii) para desarrollar un plan, y tomar las medidas necesarias para abordar estas lagunas en consulta con las organizaciones participantes.

**Resultados:**

Las principales lagunas identificadas son las siguientes (i) la falta de financiación dedicada a la vigilancia de la resistencia a los antimicrobianos; (ii) la ausencia de directrices estándar para las pruebas de susceptibilidad a los antimicrobianos; (iii) la escasez de cepas de referencia para las pruebas y la garantía de calidad; así como (iv) la ausencia de mecanismos para compartir datos. Para subsanar estas deficiencias, creamos un procedimiento operativo estándar veterinario para las pruebas de susceptibilidad a los antimicrobianos, llevamos a cabo un ejercicio de validación para identificar los problemas de aplicación del procedimiento y realizamos talleres de capacitación para los laboratorios veterinarios.**Conclusión** Las redes de vigilancia de la resistencia a los antimicrobianos dependen de la disponibilidad de pruebas precisas y de calidad controlada. Los retos identificados en la creación de una red de vigilancia integrada para la India pueden superarse mediante el desarrollo de un plan integral para mejorar la capacidad de los laboratorios en los sectores humano, veterinario y medioambiental que cuente con los fondos necesarios. Las conclusiones del estudio pueden servir de orientación a otros países de ingresos bajos y medios que se propongan crear una red similar.

## ملخص

الغرض ****تقييم مدى استعداد المعامل البيطرية في الهند للمشاركة في شبكة متكاملة لمراقبة مقاومة مضادات الميكروبات والتعامل مع الفجوات في هذه الشبكة.

الطريقة تعاون كل من المجلس الهندي للأبحاث الطبية والمجلس الهندي للأبحاث الزراعية لإنجاز ما يلي: (1) اختيار ثمانية مختبرات بيطرية ميكروبيولوجية ممثلة على الصعيد الوطني، سيتم تقييم قدرتها على المشاركة في شبكة متكاملة لمراقبة مقاومة مضادات الميكروبات باستخدام أداة قياسية؛ و(2) تحديد الفجوات في التوفير من نتائج التقييم؛ و(3) وضع خطة واتخاذ الخطوات اللازمة للتعامل مع هذه الفجوات بالتشاور مع المنظمات المشاركة.

النتائج ****كانت فجوات التوفير الرئيسية التي تم تحديدها هي: (1) نقص التمويل المخصص لمراقبة مقاومة مضادات الميكروبات؛ و(2) عدم وجود دلائل قياسية لاختبار الحساسية لمضادات الميكروبات؛ و(3) نقص في السلالات المرجعية للاختبار وضمان الجودة؛ و(4) عدم وجود آليات لمشاركة البيانات. لقد تعاملنا مع هذه الفجوات عن طريق وضع إجراء تشغيل قياسي بيطري لاختبار الحساسية لمضادات الميكروبات، عن طريق تنفيذ تمرين تحقق لتحديد مشاكل تنفيذ الإجراء، وعقد ورش عمل لبناء القدرات للمختبرات البيطرية.

الاستنتاج ****تعتمد شبكات مراقبة مقاومة مضادات الميكروبات على توافر اختبارات دقيقة ذات تحكم في الجودة. إن التحديات التي تم تحديدها في إنشاء شبكة مراقبة متكاملة للهند، يمكن التغلب عليها من خلال وضع خطة شاملة لتحسين القدرات المختبرية في القطاعات البشرية والبيطرية والبيئية المدعومة بالأموال الضرورية. قد توفر نتائج الدراسة إرشادات للدول الأخرى ذات الدخل المتوسط والدخل المنخفض والتي تخطط لتطوير شبكة مماثلة.

## 摘要

**目的:**

旨在评估印度兽医实验室在加入综合抗菌素耐药性监测网络及根据识别结果解决配置差距方面的准备工作是否到位。

**方法:**

印度医学研究理事会和印度农业研究理事会合作：(i) 选择在全国范围内具有代表性的八个兽医微生物学实验室，并使用标准化工具评估其参与综合抗菌素耐药性监测网络的能力；(ii) 根据评估结果确定在配置方面的差距；以及 (iii) 制定计划并采取必要步骤，以便与参与组织协商解决这些差距。

**结果:**

研究发现，配置方面的差距主要包括：(i) 缺乏用于抗菌素耐药性监测的专用资金；(ii) 缺乏针对抗菌素敏感试验的标准指南；(iii) 缺乏用于测试和质量控制的参考菌株；以及 (iv) 缺乏数据共享机制。通过创建适用于抗菌素敏感试验的兽医标准操作程序、开展验证工作以确定在执行该程序方面存在的问题以及为兽医实验室开设能力建设讲习班，我们成功解决了这些差距。

**结论:**

能否加入抗菌素耐药性监测网络取决于能否准确开展品质管理试验。在必要的资金支持下，通过制定一项综合计划以提高人力、兽医和环境部门的实验室能力，可以解决在为印度建立综合监测网络方面所面临的难题。该研究结果可为计划开发类似网络的其他中低收入国家提供指导。

## Резюме

**Цель:**

Оценить готовность ветеринарных лабораторий Индии принять участие в интегрированной системе эпиднадзора за устойчивостью к антимикробным препаратам и устранить выявленные пробелы в обеспечении.

**Методы:**

Индийский совет по медицинским исследованиям и Индийский совет по сельскохозяйственным исследованиям сотрудничали для того, чтобы: (i) выбрать восемь репрезентативных на национальном уровне лабораторий ветеринарной микробиологии, чья способность участвовать в интегрированной системе эпиднадзора за устойчивостью к антимикробным препаратам оценивалась с использованием стандартизированного инструмента; (ii) по результатам оценки выявить пробелы в обеспечении; (iii) разработать план и предпринять необходимые шаги для устранения этих пробелов на основе консультаций с участвующими организациями.

**Результаты:**

Были выявлены следующие основные пробелы в обеспечении: (i) отсутствие целевого финансирования в рамках эпиднадзора за устойчивостью к антимикробным препаратам; (ii) отсутствие стандартных руководящих принципов для тестирования чувствительности к антимикробным препаратам; (iii) нехватка эталонных штаммов для тестирования и обеспечения качества; (iv) отсутствие механизмов обмена данными. Мы устранили эти пробелы путем создания стандартной ветеринарной операционной процедуры для тестирования чувствительности к антимикробным препаратам, путем выполнения пробной валидации для выявления проблем с внедрением процедуры и путем проведения семинаров по созданию потенциала для ветеринарных лабораторий.

**Вывод:**

Системы эпиднадзора за устойчивостью к антимикробным препаратам зависят от наличия точных тестов, прошедших контроль качества. Проблемы, выявленные при создании интегрированной системы эпиднадзора в Индии, можно разрешить путем разработки детального плана по улучшению лабораторного потенциала в человеческом, ветеринарном и экологическом секторах, поддерживаемого необходимыми средствами. Результаты исследования могут послужить руководством для других стран с низким и средним уровнем доходов, планирующих создать аналогичную систему.

## Introduction

The misuse and overuse of antimicrobials in both humans and animals are major drivers of antimicrobial resistance.^1^^–^^3^ Several clinically important antibiotics are used extensively in food-producing animals, either for metaphylaxis, prophylaxis or promoting growth,^4^^,^^5^ and these animals and their food products are recognized as prominent routes of human exposure to foodborne pathogens. Moreover, there is a risk that resistant microbial strains or genes will be transmitted to humans and enter the environment.^5^^,^^6^ Given the lack of antimicrobial resistance surveillance systems in the veterinary sector in low- and middle-income countries, it is difficult to quantify the contribution antimicrobial use in animals makes to the emergence of drug-resistant pathogens. The establishment of surveillance systems, as part of a holistic One Health approach to public health, is therefore key to understanding the transmission of drug resistance between different health sectors and for designing interventions. Antimicrobial resistance surveillance in animals is generally inadequate both regionally and globally.^7^^,^^8^ Apart from agencies like the World Health Organization and public health bodies in Canada, the European Union, Norway and the United States of America, few organizations or countries have successfully created integrated antimicrobial resistance surveillance systems that support comprehensive antimicrobial stewardship programmes in both animals and humans and that help control antimicrobial usage and its impact on human health.^9^^,^^10^

In India, the 2017 National Action Plan stated that strengthening knowledge and evidence of antimicrobial resistance through surveillance was a strategic priority.^11^ Currently, however, surveillance in the country is limited to human health and there has been little progress towards expansion into animal or environmental health.^12^ In India, there are 17 research institutes and 19 universities operating under the animal sciences division of the Indian Council of Agricultural Research but only a handful are engaged in research into antimicrobial resistance. In 2016, the Indian Council of Medical Research entered into an agreement with the Indian Council of Agricultural Research to support collaborative research on areas of mutual interest, including antimicrobial resistance.^13^ Both organizations recognized the importance of strengthening research in, and the surveillance of, antimicrobial resistance in humans and animals and took the first steps towards developing a plan for integrated surveillance. 

**Fig. 1:**
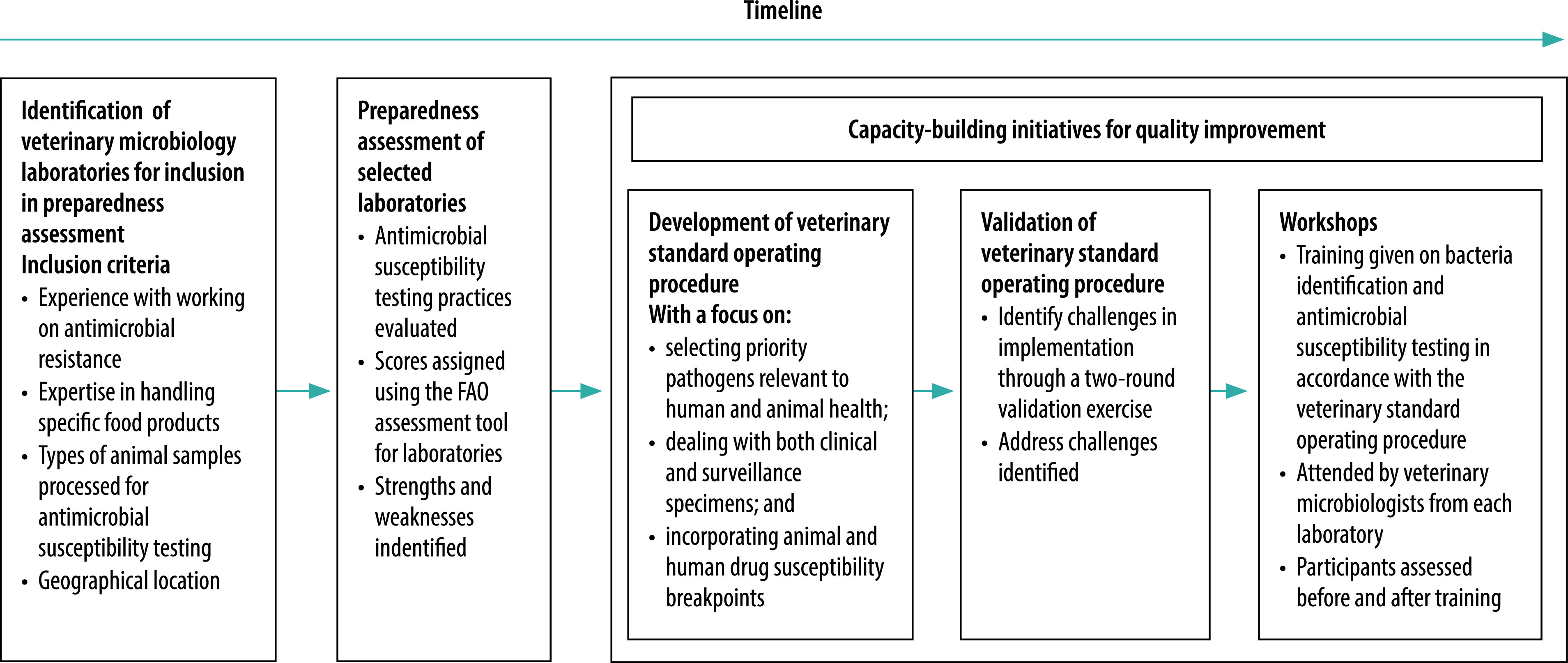
Flowchart for assessing veterinary laboratories’ preparedness for participation in a national antimicrobial resistance surveillance network and subsequent capacity-building, India, 2018

The aim of our study was, for the first time, to assess the preparedness of veterinary laboratories in India to participate in an integrated antimicrobial resistance surveillance network. We carried out a systematic assessment of laboratories and undertook capacity-building to address gaps in provision (Fig. 1).

FAO: Food and Agriculture Organization (of the United Nations).

Note: Laboratories’ preparedness for participation in a national antimicrobial resistance surveillance network was evaluated using the United Nations Food and Agriculture Organization’s (FAO) laboratory mapping tool for antimicrobial resistance, which is part of the FAO Assessment Tool for Laboratories and AMR Surveillance Systems (FAO-ATLASS).^14^

## Methods

**Fig. 2:**
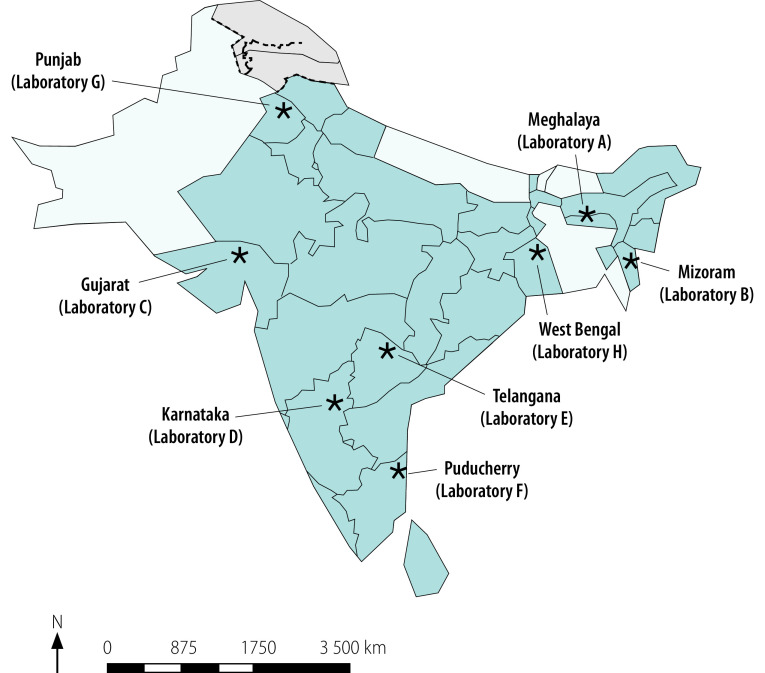
Study sites, assessment of veterinary laboratories’ preparedness for participation in a national antimicrobial resistance surveillance network, India, 2018

Notes: Study laboratories were located in the following geographical zones: (i) the north-east zone included laboratory A in Umiam, Meghalaya, and laboratory B in Aizawl, Mizoram; (ii) the west zone included laboratory C in Dantiwada, Gujarat; (iii) the south zone included laboratory D in Bengaluru, Karnataka, laboratory E in Hyderabad, Telangana, and laboratory F in Puducherry; (iv) the north zone included laboratory G in Ludhiana, Punjab; and (v) the east zone included laboratory H in Kolkata, West Bengal.

We conducted a cross-sectional survey of antimicrobial susceptibility testing at eight veterinary institutes and universities in India. Participating laboratories, which represented different geographical regions (Fig. 2), were identified through purposive sampling by the Indian Council of Agricultural Research. The inclusion criteria for laboratories covered: (i) their experience of working on antimicrobial resistance; (ii) the type and nature of animal samples they received for antimicrobial susceptibility testing; (iii) their expertise in handling specific food products; and (iv) their geographical location. We selected six veterinary laboratories in government institutes and two veterinary microbiology laboratories in academic institutions that consented to participate. Between April and July 2018, all eight laboratories were evaluated on site by a team comprising a senior microbiologist and a veterinary scientist with expertise in antimicrobial susceptibility testing and antimicrobial resistance surveillance.

### Preparedness

To assess laboratories’ preparedness to participate in a national antimicrobial resistance surveillance network, we used the United Nations Food and Agriculture Organization’s (FAO) laboratory mapping tool for antimicrobial resistance, which is part of the FAO Assessment Tool for Laboratories and AMR Surveillance Systems (FAO-ATLASS).^14^ This tool is designed to assess individual laboratories in the food and agriculture sectors in: (i) funding availability; (ii) workflow management; (iii) the availability of appropriate standard operating procedures; (iv) collaboration and partnerships with other national and international laboratories; (v) relevant publications; (vi) opportunities for staff training on antimicrobial susceptibility testing; (vii) the management of biological materials, including the mode of sample identification and tracking; (viii) the archiving of bacterial isolates and the type of inventory; and (ix) preservation methods. A score for laboratory capacity (from 0 to 100%) was derived automatically for each veterinary laboratory from the completed assessment form, with a score above 70% indicating that laboratory capacity was sufficient for participation in antimicrobial resistance surveillance.

### Standard operating procedure

Standardized laboratory practices are essential for accurately interpreting antimicrobial resistance data. In cooperation with the Indian Council of Agricultural Research and participating institutions, therefore, we developed an action plan and strategic framework for improving the capacity of veterinary laboratories to be implemented between August 2018 and September 2019. There were three steps: (i) preparation of a veterinary standard operating procedure; (ii) validation of the procedure by monitoring its implementation; and (iii) training laboratory personnel.

The veterinary standard operating procedure was devised through consultations with veterinary clinicians and microbiologists with the goal of enabling antimicrobial resistance patterns in animals and humans to be compared. The overarching aims were: (i) to standardize, and ensure the accuracy of, antimicrobial resistance data; and (ii) to ensure that veterinary isolates are appropriately tested for drugs commonly used in veterinary practice and that comparisons can be made directly with drug susceptibility breakpoints (which indicate whether or not a bacterial species is susceptible to antibacterials) in humans for the purpose of integrated surveillance.

The resulting veterinary standard operating procedure itemizes methods for bacterial isolation, for identifying species using biochemical tests and for the molecular characterization of five pathogens of public health importance. It also specifies antimicrobial susceptibility testing method and gives updated data on breakpoints for different antibiotics important for animal and human health.^15^^,^^16^ To facilitate data collection for the purpose of integrated surveillance, we harmonized breakpoints with standard operating procedures for bacteriological assessment of human clinical samples. The incorporation of breakpoints from the Clinical and Laboratory Standards Institute’s veterinary guidelines ensured that veterinary specimen isolates were interpreted correctly and were, therefore, useful for veterinary clinical practice.^17^ This standard operating procedure has been accepted by the Indian Council of Agricultural Research and the use of such standard operating procedures is now obligatory for institutions belonging to the Indian Network for Fishery and Animals Antimicrobial Resistance,^18^ which was established by the Indian Council of Agricultural Research.

#### Validation

We performed a validation exercise for the veterinary standard operating procedure to identify challenges faced by laboratories in implementing it and to address these challenges before the procedure document was finalized. Four laboratories with high scores on the preparedness assessment volunteered to participate in two rounds of validation. Laboratories were instructed to perform antimicrobial susceptibility testing and interpretation according to the procedure. In the first round, five isolates each of five pathogens were provided in 25 silica gel pouches in a blinded manner. The five pathogens, which were selected by the Indian Council of Medical Research and the Indian Council of Agricultural Research because of their importance for public health and evidence of potential transmission, were: *Escherichia coli*, *Klebsiella* spp., non-typhoidal *Salmonella*, staphylococci (four *Staphylococcus aureus* and one coagulase-negative staphylococcus) and enterococci. Four reference strains were also provided: *E. coli* ATCC 35218, *Klebsiella pneumoniae* ATCC 700603, *S. aureus* ATCC 25923 and *Enterococcus faecalis* ATCC 51299. For all five pathogens, laboratories received a list of six antibiotics for antimicrobial susceptibility testing. In the second round, the same five pathogens were provided in 13 isolates: two isolates each of *E.coli, Klebsiella*, non-typhoidal *Salmonella* and enterococci and five staphylococci isolates (three *S. aureus* and two coagulase-negative staphylococci).

Laboratories were scored on: (i) antimicrobial susceptibility testing; (ii) minimum inhibitory concentration findings; and (iii) susceptibility interpretation errors. Two points were awarded for correct organism identification: 1 point each for genus and species identification. For susceptibility testing, a maximum of 2 points were awarded per drug for the six antibiotics provided (maximum: 12 points). In the evaluation of antimicrobial susceptibility testing, the number of drugs a laboratory used to test each isolate was also considered (e.g. if a laboratory tested only four drugs, the accuracy of interpretation of susceptibility testing for a single isolate was calculated from a maximum of 8 points; four drugs by 2 points each). Susceptibility interpretation errors were recorded as no error (score: 2) or a minor (score: 1), major (score: 0) or very major (score: –1) error. A minor error occurred when a susceptible or resistant isolate was reported as intermediate or an intermediate isolate was reported as susceptible or resistant. A major error occurred when a susceptible isolate was reported as resistant and a very major error, when a resistant isolate was reported as susceptible. Following the validation exercise, we organized workshops for the veterinary institutes and universities to improve performance.

## Results

### Preparedness

The strengths and weaknesses of the eight veterinary laboratories, as evaluated using the laboratory assessment tool for antimicrobial resistance, are reported in Table 1. Scores were awarded for four areas: (i) laboratory activities; (ii) technical factors; (iii) management of data and biological materials; and (iv) quality assurance. The mean score of the laboratories across 11 categories in the four areas was 60.4% (range: 59.5–84.2). Most laboratories were strong in workflow organization (mean score: 87.5%; 95% confidence interval, CI: 77.0–97.9), antimicrobial susceptibility testing method (mean score: 80.2%; 95% CI: 72.7–87.7) and the management of biological materials (mean score: 73.3%; 95% CI: 68.4–78.2). Six of the eight laboratories reported ample availability of resources (e.g. media, reagents, equipment and facilities) for the identification and susceptibility testing of a broad range of bacterial species: they scored over 70.0%. In addition, these six laboratories routinely performed molecular characterization for over 30.0% of resistant isolates. Two laboratories (laboratories D and H) sequenced resistance genes from more than 100 isolates annually. Overall, the mean score for molecular characterization was 77.0% (95% CI: 63.3–90.7). The laboratories’ scores for data management varied widely from 55.6% to 100.0% (mean: 77.8%; 95% CI: 65.4–78.2). The assessment also found there was no mechanism for data sharing with a data collection unit: only four of the eight laboratories shared data and this sharing was irregular and partial.

**Table 1:** Assessment of veterinary laboratories’ preparedness for participation in a national antimicrobial resistance surveillance network, India, 2018

Assessment area and category	Assessment score (%)^a^												
	Geographical zone of India^b^												Mean (95% CI)
	North-east			West		South				North		East	
	Laboratory A	Laboratory B		Laboratory C		Laboratory D	Laboratory E	Laboratory F		Laboratory G		Laboratory H	
**Laboratory activities**													
Sustainability of activities	16.7	100.0		100.0		16.7	33.3	33.3		100.0		0.0	50.0 (20.4–79.6)
Workflow organization	100.0	66.7		77.8		88.9	66.7	100.0		100.0		100.0	87.5 (77.0–97.9)
Collaboration with other laboratories	75.0	91.7		50.0		66.7	83.3	66.7		83.3		83.3	75.0 (65.7–84.2)
**Technical factors**													
Bacteriology resources	94.4	83.3		83.3		72.2	72.2	44.4		94.4		55.6	74.9 (62.6–87.3)
Antimicrobial susceptibility testing methodology	96.3	77.8		63.0		92.6	77.8	71.4		77.8		85.2	80.2 (72.7–87.7)
Molecular characterization of pathogens	83.3	50.0		66.7		100.0	83.3	50.0		83.3		100.0	77.0 (63.3–90.7)
**Management of data and biological materials**													
Management of biological materials	80.0	66.7		73.3		86.7	66.7	66.7		73.3		73.3	73.3 (68.4–78.2)
Data management	77.8	55.6		88.9		77.8	100.0	55.6		66.7		100.0	77.8 (65.4–78.2)
Documentation	66.7	50.0		33.3		66.7	50.0	33.3		50.0		50.0	50.0 (41.2–58.7)
**Quality assurance**													
Methods	100.0	100.0		0.0		100.0	83.3	33.3		100.0		100.0	77.0 (50.2–103)
Staff	83.3	16.7		50.0		83.3	66.7	50.0		66.7		NA	59.5 (42.2–76.7)
**Total^c^**	**84.2**	**71.8**		**67.5**		**79.5**	**73.0**	**59.5**		**81.2**		**74.3**	**60.4 (47.6–73.1)**

CI: confidence interval; NA: not available.

^a^ Preparedness was assessed using the United Nations Food and Agriculture Organization’s (FAO) laboratory mapping tool for antimicrobial resistance, which is part of the FAO’s Assessment Tool for Laboratories and AMR Surveillance Systems; it comprises four areas with 11 categories and 40 subcategories.

^b^ The locations of the laboratories are shown in Fig. 2.

^c^ We calculated total value using the FAO’s Assessment Tool.

Five laboratories scored under 34% for the sustainability of their antimicrobial resistance assessment activities (mean: 50.0%; 95% CI: 20.4–79.6). Six of the eight scored 50% or less for documentation in the quality assurance area due to the absence of standard operating procedures (mean: 50%; 95% CI: 41.2–58.7). Only six laboratories regularly used and tested reference strains for antimicrobial susceptibility as part of quality assurance; the other two laboratories had no reference strains available. No laboratory did proficiency testing for the quality assurance of antimicrobial susceptibility testing. Two laboratories (laboratories B and H; Table 1) performed poorly on staff training (i.e. scored less than 50%) as laboratory personnel had not received any recent training on antimicrobial susceptibility testing.

### Standard operating procedure

In the first validation round for the veterinary standard operating procedure, two of the four laboratories examined (laboratories A and D; Table 2) evaluated all 25 cultures provided, whereas the other two (laboratories G and H) were able to evaluate only 22 of the 25 because of the non-revival or contamination of cultures. Laboratory D identified the highest number correctly (i.e. 90.0%; 45/50), including the genus of 100.0% (25/25) and the species of 80.0% (20/25). Laboratories A, G and H accurately identified 48.0% (24/50), 50.0% (22/44) and 75.0% (33/44) of cultures, respectively (Table 2). Most laboratories scored low on antimicrobial susceptibility testing (i.e. below 90%) due to major and minor errors. For example: (i) gentamicin-resistant *Enterococcus* spp. were reported as susceptible by one laboratory; (ii) *S. aureus* was reported as intermediate instead of susceptible by one laboratory; and (iii) colistin testing was not performed as required in the standard operating procedure by two laboratories – these two used the disk diffusion method instead of broth microdilution for colistin and vancomycin susceptibility testing, hence scored 0 points. All laboratories experienced difficulty in differentiating: (i) *S. aureus* from coagulase-negative staphylococci; (ii) typhoidal from non-typhoidal *Salmonella*; and (iii) *E. faecium* from *E. faecalis*. They all relied on molecular testing for the isolation and identification of *Salmonella* spp. because reagents for serotyping were not available.

**Table 2:** Assessment of veterinary laboratories’ ability to implement the veterinary standard operating procedure for assessing antimicrobial resistance, India, 2018–2019

Laboratory^a^	No. of cultures evaluated			% of culture (no.)									Susceptibility testing % (points/maximum points*)*^c^	
				Bacterial genus identified correctly			Bacterial species identified correctly			Culture identified correctly^b^				
	First validation round	Second validation round		First validation round	Second validation round		First validation round	Second validation round		First validation round	Second validation round		First validation round	Second validation round
**A**	25	11		76.0 (19)	100.0 (11)		20.0 (5)	81.8 (9)		48.0 (24)	90.9 (20)		80.4 (127/158)	96.0 (119/124)
**D**	25	13		100.0 (25)	100.0 (13)		80.0 (20)	100.0 (13)		90.0 (45)	100.0 (26)		88.3 (182/206)	94.9 (148/156)
**G**	22	11		72.7 (16)	72.7 (8)		27.3 (6)	54.5 (6)		50.0 (22)	63.6 (14)		71.2 (104/146)	82.3 (79/96)
**H**	22	11		86.0 (19)	90.9 (10)		63.6 (14)	72.7 (8)		75.0 (33)	81.8 (18)		76.4 (139/182)	90.2 (83/92)

^a^ The locations of the laboratories are shown in Fig. 2.

^b^ For each culture, genus identification and species identification accuracy were combined.

^c^ The accuracy of each laboratory’s interpretation of susceptibility testing was calculated as follows: (i) a maximum of 2 points was awarded for each drug tested against each bacterial isolate, where interpretation errors were categorized as no error (2 points) or a minor (1 point), major (0 points) or very major (–1 point) error; and (ii) the total number of points awarded was expressed as a percentage of the maximum possible score if all drugs tested against all isolates by that laboratory were interpreted correctly. For example, if a laboratory tested six drugs against 25 cultures, the maximum possible score would be 6 x 25 x 2 = 300 points.

Note: We evaluated the four laboratories’ ability to implement the veterinary standard operating procedure in two validation rounds.

After the first validation round, a consultative meeting was held between experts in antimicrobial susceptibility testing and veterinary microbiologists from participating laboratories and the results of the validation exercise were shared. Weaknesses identified were discussed and solutions were suggested by the experts. A second validation round was recommended. All four laboratories showed substantial improvements across all parameters in the second round (Table 2). The laboratories correctly identified *Salmonella* spp. and coagulase-negative staphylococci using the veterinary standard operating procedure. In addition, three of the four laboratories scored 90% or higher for the interpretation of antimicrobial susceptibility testing findings, compared with 71.2–88.3% in first round (Table 2).

#### Training workshops

Having identified the challenges faced by veterinary microbiologists during the validation exercise, training workshops were organized to improve antimicrobial susceptibility testing by ensuring it conformed with the veterinary standard operating procedure. The workshops were attended by two veterinary microbiologists and two laboratory staff from each of the eight participating institutes. Workshops took place at two sites to avoid overcrowding: the microbiology departments of the Christian Medical College, Vellore, and the Postgraduate Institute of Medical Education and Research, Chandigarh, respectively.

The 3-day workshops were held in September 2019, covered both theoretical and practical aspects of antimicrobial susceptibility testing and included a 2-day, hands-on workshop on bacterial identification and antimicrobial susceptibility testing as stipulated by the operating procedure. The workshop curriculum covered: (i) choosing the correct combination of drug and microbe; (ii) selecting the appropriate testing method; and (iii) guidance on the accurate reading and interpretation of susceptibility testing data. Participants were evaluated by questionnaire before and after training. Questionnaire findings showed that the proportion of participants who understood pathogen and species identification on antimicrobial susceptibility testing improved from 25% (55/220) before training to 60% (132/220) after.

## Discussion

Surveillance of antimicrobial resistance in line with the One Health concept requires the creation of integrated national networks. Most countries have pledged to set up these networks but are facing their own individual challenges.^19^ Researchers have suggested that the most common challenges are: (i) inadequate funding; (ii) poor communication between agencies; and (iii) limited participation of human and animal experts, including those in environmental health.^20^ Our collaborative study identified several factors that may hamper the creation of integrated surveillance networks in India and other low- and middle-income countries: (i) a lack of dedicated funding for antimicrobial susceptibility testing; (ii) a lack of trained staff; (iii) the absence of essential quality control measures; and (iv) a failure to take into account updated guidelines and drug susceptibility breakpoints. These factors, which have been highlighted by other researchers,^21^ affect the quality and accuracy of antimicrobial resistance data and the sustainability of surveillance. Most work on antimicrobial resistance at laboratories in our study was heavily dependent on short-term or project funding from national and international agencies. No funding for antimicrobial resistance surveillance had been received from the Indian Council of Agricultural Research or the government. These financial constraints can limit laboratory capacity, thereby compromising data quality. In addition, the constrained availability of resources was also reflected in a lack of essential reagents and a shortage of the reference strains needed for quality assurance. 

As we recognized that the lack of a veterinary standard operating procedure for antimicrobial susceptibility testing was a major deficiency in India, we developed an operating procedure for priority pathogens that incorporated national susceptibility breakpoints.^15^ In our validation exercise to determine how easily laboratories could adopt the procedure and how accurately they could apply it, all four laboratories admitted they did not follow it when identifying pathogens or interpreting the results of antimicrobial susceptibility testing. Instead, laboratories chose to follow previous procedures (e.g. by using the disk diffusion test for colistin instead of the recommended broth microdilution method), which indicated behavioural nonconformance. During the first validation round, we observed a reluctance to adopt the new procedure, which could obstruct its implementation across the country. Laboratories may, therefore, need to be convinced of its advantages. We regarded the limitations we identified through this assessment as an opportunity for improvement rather than as a setback. After laboratory staff met experts, who explained the importance of following standard operating procedures, all laboratories performed as well or better on all measures in the second validation round, which illustrates the importance of understanding laboratory staff’s concerns and explaining the usefulness of following new procedures. The failure to correctly identify susceptible or resistant isolates in the second round or to mistakenly interpret breakpoints, thereby leading to erroneous antimicrobial susceptibility testing results, indicated that laboratory staff had received inadequate training.

Our findings suggest that a national plan to build laboratory capacity is needed to ensure that the data collected by an integrated antimicrobial resistance surveillance network are of high quality (Box 1). All stakeholders should participate in the development of the plan, which should involve periodic training, quality improvement initiatives and rigorous follow-up protocols. Clearly, political commitment and sustained funding will be crucial for achieving the desired outcomes. Moreover, implementation of the plan would benefit from the support of trusted champions who can provide guidance and support across all sectors.

Box 1 **Main study findings**

- A One Health approach to antimicrobial resistance surveillance necessitates the creation of an integrated network.
- Most data on antimicrobial resistance come from human sources as veterinary and environmental sectors have limited capacity for antimicrobial susceptibility testing.
- Political commitment and leadership and multisectoral collaboration are critical for building an integrated surveillance network.
- A top-down approach may not result in multisectoral collaboration or ensure the availability of resources for an integrated network.
- The way forward is to identify and address challenges in implementation.
- Countries planning to create an integrated network can learn from others’ experience.

**Fig. 3:**
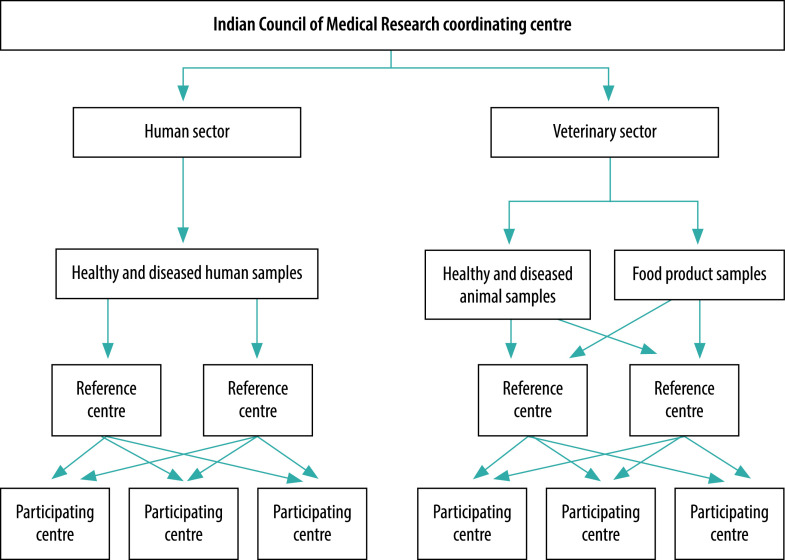
Proposed antimicrobial resistance surveillance network for India

Notes: The coordinating centre would be responsible for: (i) guideline development; (ii) development of standard operating procedures; (iii) development of training programmes; (iv) quality assurance; and (v) data analysis. Reference centres would be responsible for: (i) antimicrobial susceptibility testing; (ii) studying resistance mechanisms; (iii) conducting molecular and transmission dynamics studies; (iv) organizing training hubs; (v) data validation; and (vi) providing data to the coordinating centre and to participating centres. Participating centres would be responsible for: (i) isolating and identifying pathogens and antimicrobial susceptibility testing; (ii) storing microbes; (iii) sending isolates to reference centres; (iv) acting as training hubs; (v) undergoing training from reference centres; and (vi) providing data to reference centres.

In 2013, the Indian Council of Medical Research established a national antimicrobial resistance surveillance network for human health, which today provides 15 000 United States dollars (US$) annually to participating hospitals and has invested in capacity-building and quality assurance initiatives.^12^ We drafted a similar plan for an integrated surveillance programme that will collect comparable data from both veterinary and human sectors on five pathogens of public health importance (i.e. *E. coli*, *Klebsiella*, non-typhoid *Salmonella*, staphylococci and enterococci). We identified eight reference centres in India: four human centres for healthy and diseased human samples and four veterinary centres for healthy and diseased animal and food product samples. Three of the five pathogens will each be assessed by one human and one veterinary laboratory, whereas staphylococci and enterococci will be assessed together by the fourth human and the fourth veterinary laboratory. These reference centres will: (i) carry out antimicrobial susceptibility testing; (ii) study resistance mechanisms; (iii) conduct in-depth molecular and transmission dynamics studies; and (iv) provide training for other laboratories. In addition, between eight and 10 potential participating centres have been identified: these centres will help in sample collection and provide isolates of the five key pathogens to the relevant reference laboratories (Fig. 3). Our experience with funding of the Indian Council of Medical Research’s human antimicrobial resistance network indicates that the estimated annual cost would be US$ 55 000 for each reference centre and US$ 20 000 for each participating centre, which would cover human resources, non-recurring expenditure (e.g. for equipment), consumables, training, communication, travel, transport and other costs.

Having established a functional antimicrobial resistance surveillance system for human health, India has an opportunity to build on past experience and design an efficient integrated system that generates high-quality data on pathogens of concern for public health. In its 2017 National Action Plan,^11^ the Indian government made a commitment to support antimicrobial resistance surveillance and to strengthen laboratories in human, animal and environmental health. However, this commitment has not been translated into an implementable plan with the requisite financial resources. We hope the findings of our systematic assessment and capacity-building exercise will contribute to the development of an integrated laboratory network in India. We believe our study is an important step in the creation of a long-term, integrated, antimicrobial resistance surveillance strategy linking human and veterinary sectors in the country and hope it will provide guidance to other countries planning to develop similar strategies.
