# Corrigendum

**DOI:** 10.2471/BLT.22.100422

**Published:** 2022-04-01

In: Vijay S, Sharma M, Misri J, Shome BR, Veeraraghavan B, et al. An integrated surveillance network for antimicrobial resistance, India. Bull World Health Organ. 2021 Aug 1;99(8):562–71, 

On page 563, Fig. 2 should be as follows: 

**Fig. 2:**
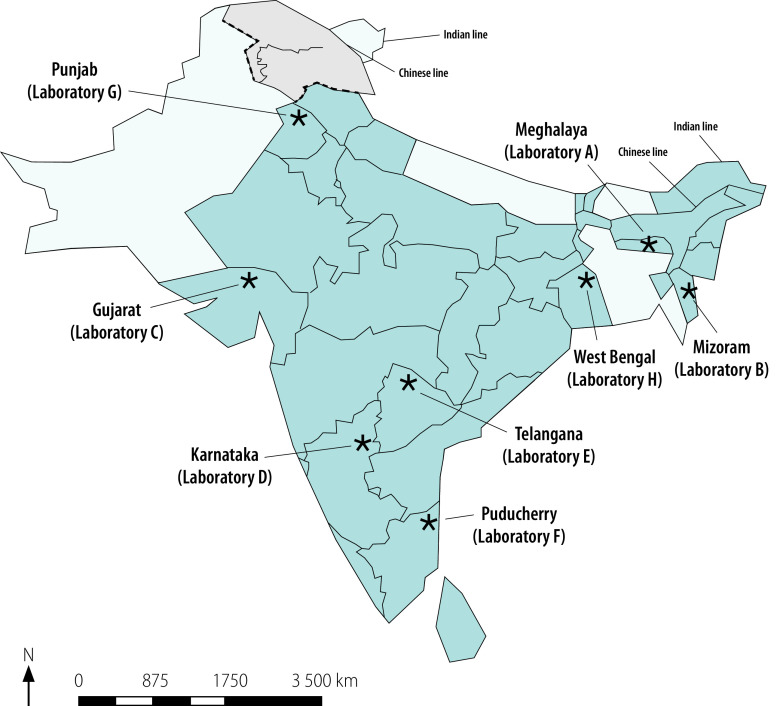
Study sites, assessment of veterinary laboratories’ preparedness for participation in a national antimicrobial resistance surveillance network, India, 2018

Notes: Study laboratories were located in the following geographical zones: (i) the north-east zone included laboratory A in Umiam, Meghalaya, and laboratory B in Aizawl, Mizoram; (ii) the west zone included laboratory C in Dantiwada, Gujarat; (iii) the south zone included laboratory D in Bengaluru, Karnataka, laboratory E in Hyderabad, Telangana, and laboratory F in Puducherry; (iv) the north zone included laboratory G in Ludhiana, Punjab; and (v) the east zone included laboratory H in Kolkata, West Bengal.

